# Linking proactive motivation to search for and presence of calling among kindergarten teachers: a variable-centered and person-centered approach

**DOI:** 10.1186/s40359-026-04359-y

**Published:** 2026-03-12

**Authors:** Wenjing Zeng, Runkai Jiao, Dandan Huang, Zhengya Teng, Feifei Li

**Affiliations:** 1 Chinese Education Modernization Research Institute of Hangzhou Normal University (Zhejiang Provincial Key Think Tank), Hangzhou, China; 2 Jing Hengyi School of Education, Hangzhou Normal University, Hangzhou, China; 3 School of Psychology, Northeast Normal University, Changchun, China; 4 College of Education, Wenzhou University, Wenzhou, China

**Keywords:** Search for calling, Presence of calling, Proactive motivation, Latent profile analysis

## Abstract

Teachers’ calling is a key psychological resource in education, encompassing search and presence. Based on the Proactive Motivation Model, calling is influenced by “can do,” “reason to,” and “energized to” motivational states. While past research has linked individual motivational factors to calling presence, how multiple motivational states jointly influence both calling dimensions remains unclear. This study investigated how career adaptability (can do), organizational identification (reason to), and positive affect (energized to) predict the presence of and search for a calling. Using complementary variable- and person-centered approaches, data were analyzed from 1674 kindergarten teachers in China. Variable-centered findings showed that all three motivational states uniquely predicted the presence of calling. For calling search, only career adaptability and organizational identification were positive predictors; positive affect was non-significant. Person-centered results revealed four calling profiles: High Maintenance in Calling, Low Persistence in Calling Keep Unchanged, Moderate Development in Calling, and Emergent Development in Calling. Career adaptability positively influenced all profiles, whereas organizational identification and positive affect differentiated only specific profiles. These findings highlight the multidimensional motivational basis of calling and reveal distinct pathways in teachers’ calling development.

## Introduction

Early childhood teachers play a pivotal role in children’s cognitive and socio-emotional development. Their work involves high emotional demands and low societal recognition, increasing risks of burnout and turnover [48, 53, 55]. Within this challenging context, calling has emerged as a critical psychological resource. Empirical evidence demonstrates that perceiving a calling is linked to lower burnout and higher job satisfaction, well-being, and organizational commitment among educators [25, 29, 55]. Yet, vocational experience involves not only the presence of a calling but also actively searching for one; the latter represents a critical yet underexamined aspect of vocational development [11, 17]. This study focuses on both the presence of and search for a calling among kindergarten teachers and examines their predictors.

To understand the psychological drivers of kindergarten teachers’ calling, this study draws on the Proactive Motivation Model (PMM; [41]), which explains how individuals initiate and sustain proactive goals—self-initiated, future-oriented efforts to create meaningful change. A key type of such goal is achieving better person–environment fit, which aligns closely with calling, as both involve self-set aims marked by alignment between personal interests, values, abilities, and social needs within one’s role [5, 8, 16]. The PMM identifies three proximal motivational states that facilitate both the formation and persistence of such goals: “can do”, “reason to”, and “energized to”. Grounded in this theoretical perspective, the present study examines how these motivational states—specifically career adaptability (can do), organizational identification (reason to), and positive affect (energized to)—predict both the search for and presence of calling among kindergarten teachers.

Research on calling has mainly used variable-centered approaches to identify predictors such as trait gratitude [33], psychological need satisfaction and frustration [35], and expected-perceived support fit [58]. These studies assume population homogeneity that a single set of parameters can describe the entire population [24]. However, person-centered research challenges this view by revealing distinct, meaningful patterns in how calling presence and search co-occur (e.g., Li et al., [32]. Moreover, the factors underlying the emergence of these profiles and the motivational drivers of different calling configurations remain insufficiently understood. To address this gap, this study integrates variable-centered and person-centered approaches, which are complementary methods and together enable a more comprehensive understanding of the same phenomenon [24], to examine the effects of three proactive motivations on the calling search and presence.

### Search for and presence of calling

Calling is a psychological orientation toward work defined by a sense of transcendent purpose, alignment with life meaning, and other-oriented intentions [13]. It consists of two components: search for calling and presence of calling [9]. Search for calling refers to the active effort to identify or deepen engagement in a meaningful career through self-reflection, internal exploration, and environmental feedback [17, 22]. Presence of calling reflects the extent to which individuals currently experience their work as purposeful and personally significant, marked by commitment, clarity, and fulfillment [9, 16]. For most people, calling is not an innate certainty but develops dynamically through personal growth and work experiences. It is an ongoing process of meaning-making rather than a one-time discovery [7]. Even after a calling is perceived, individuals often continue searching to sustain, refine, or adapt it over time [9].

Theoretical and empirical research indicates that kindergarten teachers’ calling is not uniform but varies in distinct latent profiles. At its core, calling reflects an individual’s pursuit and experience of life meaning [9]. The process of meaning-making involves a dynamic between the search for and presence of meaning in life. The search for meaning drives individuals to find and develop a clearer purpose and greater sense of coherence. Through this seeking process, they are more likely to achieve a strong perception of meaning. While an intensive presence of meaning may weaken their efforts in searching for meaning, a new and higher-order meaning source can re-activate the motivation to seek [31]. This dynamic interplay theoretically allows for the coexistence of different configurations, such as individuals with high meaning presence but low search, as well as those exhibiting high levels of both seeking and presence. Empirical studies on meaning in life have confirmed such heterogeneous profiles in the general population [6, 36], supporting the likelihood of similar heterogeneity in the calling domain.

Direct empirical support for these heterogeneous profiles comes from person-centered studies on calling search and presence. Li et al., [32] conducted a latent profile analysis among kindergarten teachers and identified distinct subgroups, including “actively maintaining calling” (high search, high presence) and “unsustainable calling” (high presence, low search). Further corroborating these findings, Zhou [57], examined the fit between search for and presence of calling and identified four distinct configurations in the scientific and technological R&D employees: low search–low presence, high search–high presence, high search–low presence, and low search–high presence. Distinct profiles predicted key work-related outcomes differently, with the high search–high presence and low search–high presence patterns linked to greater job satisfaction, work meaning, and innovative behavior compared to other configurations [32, 57].

This evidence highlights the limitations of traditional variable-centered approaches, which assume sample homogeneity and estimate all cases with a single set of parameters. This variable-centered approach overlooks the heterogeneity existing in real-world contexts, thereby oversimplifying reality [49]. Person-centered methods like latent profile analysis are better suited to capture such heterogeneity by identifying meaningful subgroups based on distinct configurations of individual characteristics, thereby offering a more nuanced and realistic understanding.

### Proactive motivation model

The Proactive Motivation Model [41] provides a comprehensive framework for understanding self-initiated, goal-driven processes, involving both the generation of proactive goals and the sustained effort required to realize them. This model conceptualizes proactive goals along two core dimensions: (1) a future-oriented intended state (e.g., achieving greater person–environment fit in one’s work); and (2) a locus of change that may be directed toward the self (e.g., cultivating competencies or refining personal values) or toward the situation (e.g., redesigning job tasks or influencing organizational systems). Calling aligns closely with this conceptualization: it is inherently self-initiated, future-oriented, and change-oriented. Individuals typically pursue and develop their calling volitionally, guided by deeply held values, inner interests, or passion [11, 15]. They envision and actively strive toward a future work state that is deeply meaningful and identity-congruent [51]. The change process may target the self (e.g., developing skills or values to align with a vocation) or the situation (e.g., reshaping job tasks to express purpose), always aiming to transform current reality for a more meaningful person–environment fit [12]. Moreover, calling deepens person-environment fit from instrumental compatibility (e.g., matching skills to tasks) to meaningful compatibility, where work expresses personal values, identity, and purpose. Unlike traditional proactive goals which instrumentally target discrete and observable actions to improve performance, calling is an existential and identity-based goal centered on one’s true self through work, a deeper sense of purpose, and contribution beyond daily tasks.

The Proactive Motivation Model posits that proactive goal processes are jointly sustained by three distinct motivational states: “can do”, “reason to”, and “energized to” [41]. Can do motivation reflects an individual’s belief in their ability to execute a specific proactive behavior, grounded in self-efficacy, perceived control, and low anticipated costs. Reason to motivation refers to autonomous motivations underlying proactive engagement, addressing why individuals choose or persist in pursuing particular proactive goals. Energized to motivation reflects high-arousal positive affective states—such as enthusiasm and excitement—that energize goal-directed behavior, making individuals more likely to initiate and sustain proactive efforts. These states are interrelated and collectively represent the proximal motivational architecture that enables individuals to generate and strive toward proactive goals. Recent empirical studies across diverse domains have modeled multiple PMM motivational states as parallel-yet-integral predictors of various proactive outcomes, such as proactive service performance and pro-environmental behavior [19, 59].

### The effect of proactive motivation on calling

According to the PMM, when individuals feel capable, have compelling reasons, and/or possess sufficient energetic resources in a given domain, they are more likely to set and pursue proactive goals—such as person-environment fit, a central aspect of calling [15].

Career adaptability is a key self-regulatory capacity that enables individuals to manage career tasks, transitions, and challenges, commonly viewed as a “can do” motivation [20, 47]. It comprises four components: concern (planning for one’s vocational future), control (making career decisions), curiosity (exploring possible selves), and confidence (pursuing career goals persistently). These resources support proactive self-exploration, clarify personal values, and strengthen the fit between work and identity, which are foundations for discerning and developing a calling [22]. A meta-analysis by Rudolph et al., [44] identifies calling as a key outcome of career adaptability within career construction theory. As a core career meta-competency, career adaptability facilitates both the pursuit and experience of calling [22], with empirical evidence showing its positive effects on calling [21, 58]. Thus, kindergarten teachers with higher career adaptability are more likely to actively search for or perceive a strong sense of calling.

Organizational identification reflects a “reason to” motivation [3, 41], defined as the extent to which individuals internalize an organization’s values and goals into their self-concept [34]. Research shows it positively predicts calling [34, 60]. Strong identification fosters a heightened sense of belonging and responsibility, motivating individuals to seek meaningful ways to contribute, such as aligning personal strengths with organizational aims. It also promotes internalization of shared values and collective goals, enhancing perceived meaningfulness and reinforcing the belief that one’s work serves a broader purpose. Since the pursuit of self-meaning and prosocial contributions is central to calling search, and the perception of these aspects is key to calling presence [9], organizational identification supports both. Clarifying identity—answering “Who am I?”—is foundational to deeper questions of purpose like “Why am I here?” [42], and calling development is a process of self-meaning construction [22]. A clear self-concept is essential for identifying and developing a calling [17]. Therefore, organizational identification is expected to positively predict both calling search and presence among kindergarten teachers.

Positive affect represents an “energized to” motivational state [41] and has been widely linked to calling. According to the broaden-and-build theory of positive emotions [18], it broadens thought-action repertoires, fostering creativity and exploratory behavior, and helps build enduring resources such as resilience and social connections. The search for a calling is cognitively and emotionally demanding, requiring sustained effort, reflection, and engagement [11]. Positive affect could support this process by enhancing cognitive openness, reducing defensiveness, and encouraging active meaning-seeking and self-discovery. It also helps individuals connect daily experiences to broader existential meanings, facilitating the perception of life purpose and calling [10, 28]. Empirical findings show that individuals who experience greater positive affect at work—such as enthusiasm—are more likely to report a stronger sense of calling [23, 29]. Hence, kindergarten teachers with frequent positive affect are expected to pursue and perceive calling more strongly.

### The present study

Building on the existing literature, this study applies the PMM to propose an integrated model in which career adaptability (“can do”), organizational identification (“reason to”), and positive affect (“energized to”) serve as key motivational resources that jointly predict both the search for and presence of calling. To rigorously test our hypotheses, we employ a novel dual-analytic strategy that combines variable-centered and person-centered approaches—using the former to identify generalizable patterns and the latter to capture meaningful heterogeneity across individuals—thereby offering a more nuanced and empirically grounded understanding of how proactive motivation relates to calling.

First, from a variable-centered perspective (e.g., multiple regression), this study assesses the extent to which these three proactive motivational states jointly and independently predict variance in calling search and presence across the kindergarten teacher sample. Based on abovementioned evidence, we assume that career adaptability, organizational identification, and positive affect are each positively related to both calling search and presence.

Second, to capture potential heterogeneity in vocational experiences, person-centered analyses using latent profile analysis (LPA) will identify distinct subgroups of teachers based on their concurrent levels of calling search and presence. Drawing on previous research identifying heterogeneous calling profiles [32, 57], we hypothesize at least two distinct calling profiles: one characterized by high calling presence and low calling search, and another by high calling presence and high calling search.

We then test a predictive model to examine how the three proactive motivational states predict membership in these profiles. Although no prior study has investigated the predictors of calling profiles, we derive exploratory hypotheses based on the theoretical and empirical evidence described above. Existing evidence demonstrates that these motivational states are positively associated with both calling search and presence (e.g. [29, 34, 58]), . Therefore, we hypothesize that higher levels of career adaptability, organizational identification, and positive affect will significantly increase the likelihood of belonging to the high–high profile (characterized by high calling presence and high calling search) relative to other profiles, such as the high presence–low search profile.

## Method

### Participants and procedure

This study used a purposive convenience sampling approach to recruit kindergarten teachers in China through the National Kindergarten Principals Training Center under the Ministry of Education, a leading institution for principal professional development. Participating principals were invited to distribute the survey link to teachers in their kindergartens. To ensure regional diversity, participants were recruited from eastern (e.g., Beijing, Tianjin, Shanghai, Hebei), central (e.g., Anhui, Jilin), and western (e.g., Yunnan, Shaanxi, Gansu) provinces and municipalities, aiming to capture a representative sample across diverse socioeconomic and geographic contexts. Before data collection, all teachers provided informed consent and were informed that participation was voluntary, anonymous, and strictly for research purposes. Only those who consented were included.

A total of 2,261 teachers completed the online questionnaire. After excluding responses with excessively short completion times (less than two seconds per item on average; [27]), uniform responding, or missing data on entire variables, 1,674 valid responses were retained. The final sample had a mean age of 32.53 years (*SD* = 8.73, range: 19–64). In terms of education, 936 (55.9%) held a bachelor’s degree or higher, 731 (43.7%) had senior high school or postsecondary specialized education, and 7 had missing data. Regarding teaching experience, 419 (25.0%) had ≤ 3 years, 569 (34.0%) had 4–9 years, 681 (40.7%) had ≥ 10 years, and 5 had missing data. As for kindergarten type, 1,243 (74.3%) worked in public kindergartens and 431 (25.7%) in private ones. Geographically, 530 (31.7%) were from eastern, 674 (40.3%) from central, and 470 (28.1%) from western regions.

### Measures

#### Calling

This study employed the Chinese adaptation [50] of the Brief Calling Scale (BCS; [9]) to measure kindergarten teachers’ search for and presence of a calling. The scale includes the Search subscale and the Presence subscale, each with two items rated on a 5-point Likert scale (1 = “not at all true of me”, 5 = “completely true of me”). Higher scores indicate greater effort in seeking a calling and a stronger sense of calling. In this study, inter-item correlations were *r* = 0.77 for the Search subscale and *r* = 0.74 for the Presence subscale.

#### Career adaptability

Career adaptability was measured using the Career Adapt-Abilities Scale-Short Form (CAAS-SF; [38]), a 12-item instrument assessing four dimensions: concern, control, curiosity, and confidence. Each dimension has three items rated on a 5-point Likert scale (1 = “not strong”, 5 = “strongest”). Higher scores indicate greater readiness, proactivity, confidence, and flexibility in managing career development, particularly amid change or uncertainty. This scale has been adapted and validated in Chinese populations, including educators, demonstrating good reliability and validity (e.g., Zhang et al., [54], Zhao [56], . In this study, Cronbach’s alpha was 0.93 for the total scale and 0.78, 0.73, 0.76, and 0.83 for the four dimensions (concern, control, curiosity, and confidence), respectively.

#### Organizational identification

Organizational identification was measured using the 6-item Organizational Identification Questionnaire adapted by Li et al., [30] from Mael and Ashforth’s [37] original scale. It assesses the extent to which individuals identify with and feel belonging to their organization. Li et al., [30] found good reliability and close association with affective commitment in Chinese teachers. Responses were given on a 5-point Likert rating scale (1 = “strongly disagree”, 5 = “strongly agree”), with higher scores indicating greater organizational identification. In the current study, Cronbach’s alpha was 0.87.

#### Positive affect

Positive affect was measured using the Positive Affect subscale of the Positive and Negative Affect Schedule (PANAS) adapted by Qiu et al., [43]. This subscale assesses the frequency of nine positive emotions (e.g., active, excited, and proud) over the past three months. Items were rated on a 7-point Likert scale (1 = “never”, 7 = “always”), with higher scores indicating greater positive affect at work in the recent past. In this study, Cronbach’s alpha for this subscale was 0.96.

### Statistical analysis

We analyzed the data in three steps. First, we conducted preliminary analyses. To assess potential common method bias (CMB), we applied two recommended confirmatory factor analytic (CFA) approaches implemented in Mplus 8.3, including Harman’s single-factor test and controlling for the effects of an unmeasured latent methods factor (ULMC) technique [46]. In the single-factor CFA, all items from the five constructs (i.e., career adaptability, organizational identification, positive affect, search for calling, and presence of calling) were loaded onto one latent factor. In the ULMC approach, we compared our five-factor measurement model with a nested model that added an orthogonal method factor. All items were allowed to load simultaneously on both their respective theoretical factors and this common method factor. Model fit was evaluated by the Comparative fit index (CFI), the Tucker-Lewis Index (TLI), the Root Mean Square Error of Approximation (RMSEA), and the Standardized Root Mean Square Residual (SRMR). CMB was considered likely if CFI/TLI exceeded 0.90 and RMSEA/SRMR fell below 0.08 in the single-factor CFA. Moreover, when adding the method factor on the five-factor model, a meaningful improvement in fit (ΔCFI ≥ 0.010; ΔRMSEA ≥ 0.015; [4]) would signal CMB. Descriptive statistics and bivariate correlations were computed in SPSS 26.0 to examine variable distributions and relationships.

Second, we used structural equation modeling (SEM) in Mplus 8.3 to examine how career adaptability, organizational identification, and positive affect predict search for and presence of calling. We specified and evaluated the measurement model before testing structural paths. Search for calling and presence of calling were each measured with two indicators, positive affect with nine items, organizational identification with six items, and career adaptability with its four dimensions (concern, control, curiosity, confidence). After achieving acceptable fit (CFI > 0.90, TLI > 0.90, RMSEA < 0.08, SRMR < 0.08; [26]), we added structural paths with the three motivational variables as predictors and the two calling variables as outcomes.

Third, we conducted latent profile analysis (LPA) in Mplus 8.3 to identify distinct calling profiles and examine how proactive motivation relates to these profiles. The scores of the search for and presence of calling served as two continuous indicators in identifying the calling profiles. We compared 1- to 5-profile models using multiple fit indices: Bayesian Information Criterion (BIC) and adjusted BIC (aBIC)—lower values indicate better fit; Entropy—higher values (> 0.80) reflect more precise classification; the adjusted Lo-Mendell-Rubin Test (LMRT) and Bootstrapped Likelihood Ratio Test (BLRT)—significant *p*-values suggest the k-profile model fits better than the (k − 1)-profile model [45]. We also considered theoretical meaningfulness and profile parsimony. For example, each retained profile should constitute more than 5% of the total sample size, and there should be clear qualitative distinctions among the profiles, rather than merely quantitative differences [45]. After determining the optimal solution, we used multinomial logistic regression with the R3STEP procedure in Mplus 8.3 [2] to assess how career adaptability, organizational identification, and positive affect predicted profile membership, controlling for demographic covariates (education, tenure, kindergarten type, region).

## Results

### Preliminary analysis

This study utilized the Harman’s single-factor test and ULMC technique to assess common method bias. The single-factor model showed poor fit (*χ*^2^ = 12351.427, *df* = 434, CFI = 0.572, TLI = 0.542, RMSEA = 0.128, SRMR = 0.135), providing initial evidence against severe common method variance. Furthermore, the more robust ULMC approach was applied. The fit of the five-factor measurement model without the method factor was good: *χ*^2^ = 2554.301, *df* = 417, CFI = 0.946, TLI = 0.939, RMSEA = 0.055, SRMR = 0.030. The inclusion of the method factor did not lead to a significant improvement in model fit (ΔCFI = 0.006, ΔTLI = 0.001, ΔRMSEA < 0.001), further indicating no substantial common method bias.

Descriptive statistics and correlations are presented in Table 1. Search for and presence of calling were significantly positively correlated (*r* = 0.37, *p* < 0.001), and both were significantly associated with career adaptability, organizational identification, and positive affect (*p* < 0.001). Tenure, kindergarten nature, and geographical region were correlated with one or both calling dimensions; educational level was not. These variables were controlled in subsequent variable-centered and person-centered analyses.

**Table 1:** Descriptive statistics and correlations

	*M*	*SD*	1	2	3	4	5
1.Search for calling	3.78	1.02	-				
2.Presence of calling	4.13	0.77	0.37^***^	-			
3.Career adaptability	4.22	0.52	0.22^***^	0.52^***^	-		
4.Organizational identification	4.52	0.55	0.15^***^	0.46^***^	0.45^***^	-	
5.Positive affect	5.57	1.05	0.12^***^	0.42^***^	0.53^***^	0.44^***^	-
6.Tenure 1	-	-	0.03	-0.10^***^	-0.04	-0.16^***^	0.05
7.Tenure 2	-	-	-0.02	0.09^***^	-0.01	0.11^***^	-0.11^***^
8.Education	-	-	-0.02	0.02	0.03	0.02	-0.08^**^
9.Kindergarten nature	-	-	0.05^*^	0.09^***^	0.02	0.06^*^	-0.06^*^
10.Zone 1	-	-	-0.01	0.08^**^	-0.04	0.01	-0.04
11.Zone 2	-	-	-0.01	0.07^*^	0.11^***^	0.05^*^	0.17^***^

*N* = 1674. ^*^p < 0.05, ^**^p < 0.01, ^***^p < 0.001. Tenure 1 (1 = ≤ 3 years) and Tenure 2 (1 = ≥ 10 years) were dummy variables transformed by tenure, in which 4 ~ 9 years as the reference base. Education: 0 = high school or postsecondary specialized college education; 1 = undergraduate or postgraduate education. Kindergarten nature: 0 = private kindergarten; 1 = public kindergarten. Zone 1 (1 = eastern area) and Zone 2 (1 = central area) were dummy variables transformed by geographical region, in which the western area as the reference base

### Profiles of calling

Table 2 presents model fit indices for solutions with two to five profiles. AIC, BIC, and aBIC decreased steadily as profile count increased. Entropy exceeds 0.80 for the four- and five-profile models, indicating high classification accuracy. Significant LMRT and BLRT results show that the four-profile model fits better than the three-profile model, and the five-profile model better than the four-profile model. Specifically, compared to the three-profile solution, the four-profile model added a qualitatively distinct profile characterized by medium-high calling presence and low calling search (*M*_presence_ = 4.31, *M*_search_ = 1.49). However, the five-profile model showed no meaningful improvement in entropy over the four-profile model. Moreover, its fifth profile (*M*_presence_ = 4.05, *M*_search_ = 2.07) closely resembles an existing profile in the four-profile solution (*M*_presence_ = 4.31, *M*_search_ = 1.49), and constitutes only 4% of the total sample—below the 5% threshold. This suggests quantitative rather than qualitative differences. Considering model parsimony and theoretical meaningfulness, the four-profile model is preferred. In this model, average posterior probabilities of class membership in the domain profile ranged from 0.941 to 0.988, and average cross-probabilities are below 0.05 (ranging from 0.012 to 0.041), supporting strong classification accuracy. Overall, the four-profile model is selected as the final and optimal solution of calling.

**Table 2:** Fit results and class sizes for the latent profile analysis (*N* = 1674)

Model	LL	FP	AIC	BIC	aBIC	Entropy	LMRT (*p*)	BLRT (*p*)	Proportion of latent profile
2 Profiles	−4193.40	7	8400.81	8438.77	8416.53	0.54	< 0.001	< 0.001	0.42/0.58
3 Profiles	−3884.24	10	7788.49	7842.72	7810.95	0.77	< 0.001	< 0.001	0.35/0.57/0.08
4 Profiles	**−**3560.15	13	7146.30	7216.80	7175.50	0.94	**< **0.001	**< **0.001	0.09/0.28/0.41/0.23
5 Profiles	−3375.85	16	6783.69	6870.46	6819.63	0.94	< 0.01	< 0.001	0.04/0.05/0.39/0.22/0.29

*LL *Model Log-Likelihood, *FP *Free Parameters, *AIC *Akaike Information Criterion, *BIC*  Bayesian Information Criterion, *aBIC *the Sample-adjusted Bayesian Information Criterion, *LMRT* (*p*) *p*-Value for the adjusted Lo-Mendell-Rubin test, *BLRT* (*p*) *p*-Value for the Bootstrapped Likelihood Ratio test

**Fig. 1:**
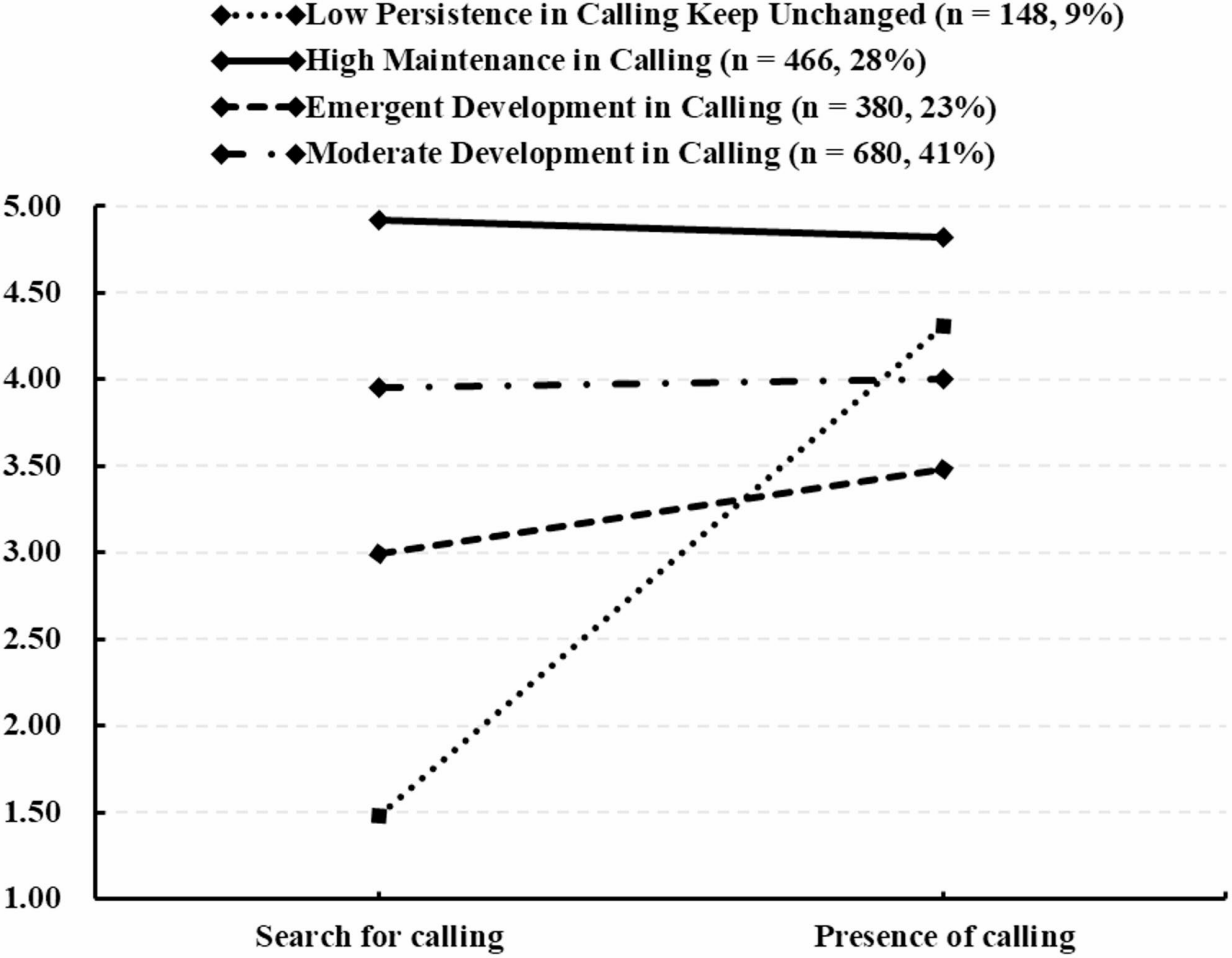
Mean scores in the indicators search for and presence of calling for the four-profile solution

Figure 1 present the mean levels of two calling variables in the four-profile solution. Drawing on prior research [32] and the theoretical roles of calling search and presence in vocational identity development [9], the four profiles are labeled as follows. The first group shows high calling presence (*M* = 4.83, *SE* = 0.02) and high calling search (*M* = 4.92, *SE* = 0.01), reflecting active efforts to sustain calling intensity; it is labeled the “High Maintenance in Calling” group (*n* = 466, 28%). The second group reports medium-high calling presence (*M* = 4.31, *SE* = 0.08) but low calling search (M = 1.49, SE = 0.04), indicating a sense of calling without further exploration to maintain it; given that calling may decline under pressure [52], this group is labeled the “Low Persistence in Calling Keep Unchanged” group (*n* = 148, 9%). The third group shows medium-high levels of both calling presence (*M* = 4.00, *SE* = 0.02) and calling search (*M* = 3.95, *SE* = 0.01), suggesting ongoing enhancement through active exploration; it is labeled the “Moderate Development in Calling” group (*n* = 680, 41%). The fourth group has medium levels of calling search (*M* = 3.00, *SE* = 0.02) and calling presence (*M* = 3.48, *SE* = 0.04), reflecting an emergent developmental state; therefore, it is labeled the “Emergent Development in Calling” group (*n* = 380, 23%).

### Variable-centered analysis: impact of proactive motivation on calling

**Fig. 2:**
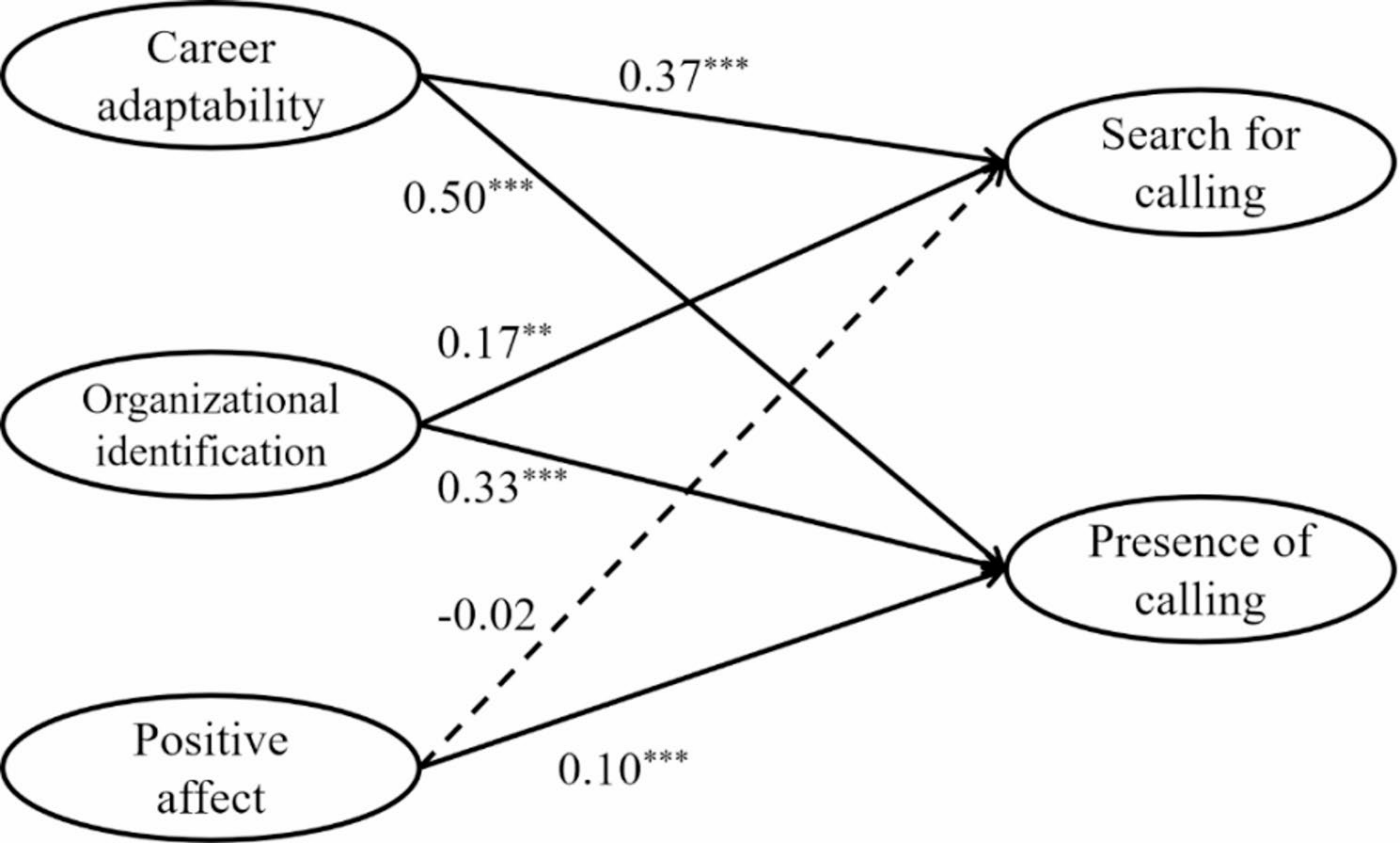
Path coefficients between proactive motivation and career calling. Note. *N* = 1674. * *p* < 0.05, ** *p* < 0.01, *** *p* < 0.001. The measurement model (including latent variable indicators) and control variables—tenure (two dummy variables: Tenure 1 [1 = ≤ 3 years], Tenure 2 [1 = ≥ 10 years]), kindergarten type (0 = private), and region (two dummy variables: Zone 1 [1 = eastern], Zone 2 [1 = central])—are omitted from the figure for clarity. All coefficients are unstandardized; dotted lines indicate non-significant paths

The hypothesized structural equation model was tested to examine the effects of three proactive motivational states (i.e., career adaptability, organizational identification, and positive affect) on search for and presence of calling. First, we established the measurement model, then extended it by adding structural paths from the three predictors to both calling dimensions, along with demographic controls, to form the full structural model. This measurement model demonstrated acceptable fit, *χ*^2^ = 1306.981, *df* = 220, CFI = 0.950, TLI = 0.943, RMSEA = 0.054, SRMR = 0.027. The structural model also fit well, *χ*^2^ = 2666.241, *df* = 325, CFI = 0.928, TLI = 0.919, RMSEA = 0.066, SRMR = 0.047. Path coefficients are shown in Table 3 and Fig. 2.

**Table 3:** Path coefficients between proactive motivation and career calling

	Search for calling			Presence of calling		
	*B*	*SE*	95% CI	*B*	*SE*	95% CI
Tenure 1	0.13^*^	0.06	[0.01, 0.25]	-0.03	0.04	[-0.11, 0.05]
Tenure 2	-0.03	0.06	[-0.15, 0.09]	0.09^*^	0.03	[0.02, 0.16]
Kindergarten nature	0.11	0.06	[-0.01, 0.24]	0.10^**^	0.04	[0.03, 0.17]
Zone 1	0.11	0.07	[-0.03, 0.24]	0.16^***^	0.03	[0.09, 0.23]
Zone 2	0.08	0.06	[-0.05, 0.21]	0.12^**^	0.03	[0.05, 0.18]
Career adaptability	0.37^***^	0.07	[0.24, 0.50]	0.50^***^	0.05	[0.41, 0.59]
Organizational identification	0.17^**^	0.06	[0.05, 0.29]	0.33^***^	0.05	[0.24, 0.42]
Positive affect	-0.02	0.03	[-0.09, 0.05]	0.10^***^	0.02	[0.05, 0.14]

*N* = 1674. ^*^p < 0.05, ^**^p < 0.01, ^***^p < 0.001. Tenure 1 (1 = ≤ 3 years) and Tenure 2 (1 = ≥ 10 years) were dummy variables transformed by tenure, in which 4 ~ 9 years as the reference base. Kindergarten nature: 0 = private kindergarten; 1 = public kindergarten. Zone 1 (1 = eastern area) and Zone 2 (1 = central area) were dummy variables transformed by geographical region, in which the western area as the reference base

As shown, career adaptability (*B* = 0.50, *p* < 0.001), organizational identification (*B* = 0.33, *p* < 0.001), and positive affect (*B* = 0.10, *p* < 0.001) were all positively associated with presence of calling. Only career adaptability (*B* = 0.37, *p* < 0.001) and organizational identification (*B* = 0.17, *p* = 0.006) significantly predicted search for calling; positive affect did not (*B* = -0.02, *p* = 0.553).

To clarify the relative strength of effects, we compared unstandardized path coefficients. For presence of calling, career adaptability exerted a stronger effect than both organizational identification (Δ*B* = 0.17, *p* = 0.025) and positive affect (Δ*B* = 0.41, *p* < 0.001), and organizational identification was stronger than positive affect (Δ*B* = 0.24, *p* < 0.001). For search for calling, career adaptability showed a non-significant effect compared to organizational identification (Δ*B* = 0.20, *p* = 0.064), and both outperformed positive affect (*p*s < 0.05).

When comparing each predictor across calling dimensions, career adaptability did not differ significantly in its effects on search versus presence (Δ*B* = 0.14, *p* = 0.050). In contrast, organizational identification (Δ*B* = 0.16, *p* = 0.002) and positive affect (Δ*B* = 0.12, *p* < 0.001) had significantly stronger effects on presence than on search. 

### Person-centered analysis: impact of proactive motivation on calling profiles

**Fig. 3:**
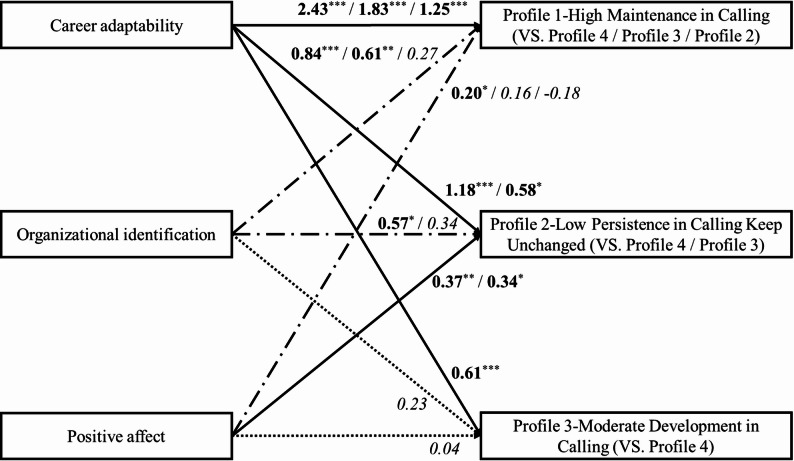
Motivational predictors of calling profile membership and developmental pathways. Note. *N* = 1674. * *p* < 0.05, ** *p* < 0.01, *** *p* < 0.001. This figure summarizes the multinomial logistic regression results (R3STEP) examining how career adaptability (“can do”), organizational identification (“reason to”), and positive affect (“energized to”) predict the likelihood of belonging to each calling profile. All coefficients are unstandardized. Solid lines indicate that all effects of this predictor across profile comparisons are statistically significant (*p* < 0.05). Dash-dotted lines (long dashes alternating with dots) indicate that only some effects are significant; the specific significant paths are noted by bolded numbers. The group following “VS.” serves as the reference category in each comparison. Profile labels: Profile 1 = High Maintenance (high presence, high search); Profile 2 = Low Persistence (medium-high presence, low search); Profile 3 = Moderate Development (medium-high presence, medium-high search); Profile 4 = Emergent Development (medium presence, medium search). For complete OR values and significance levels, see Table 4

This study used the R3STEP method in LPA to perform multinomial logistic regression, examining how proactive motivational states (i.e., career adaptability, organizational identification, and positive affect) predicted teachers’ likelihood of belonging to each of the four calling profiles, while controlling for tenure, kindergarten nature, and geographical region. Table 4 and Fig. 3 present the path coefficients.

**Table 4:** R3STEP results for the effect of proactive motivation on calling profiles

	Profile 1 VS. Profile 4		Profile 2 VS. Profile 4		Profile 3 VS. Profile 4	
	*B*	*OR*	*B*	*OR*	*B*	*OR*
Tenure 1	0.53 ^*^	1.70	0.13	1.14	0.45 ^*^	1.56
Tenure 2	0.36	1.44	0.51 ^*^	1.67	−0.09	0.92
Kindergarten nature	0.54 ^*^	1.71	0.30	1.36	0.32	1.38
Zone 1	0.40 ^*^	1.49	0.46 ^*^	1.59	−0.02	0.99
Zone 2	0.30	1.35	0.45 ^*^	1.57	0.14	1.16
Career adaptability	2.43 ^***^	11.37	1.18 ^***^	3.26	0.61 ^***^	1.83
Organizational identification	0.84 ^***^	2.31	0.57 ^*^	1.76	0.23	1.26
Positive affect	0.20 ^*^	1.22	0.37 ^**^	1.45	0.04	1.04

	Profile 1 VS. Profile 3		Profile 2 VS. Profile 3		Profile 1 VS. Profile 2	
	*B*	*OR*	*B*	*OR*	*B*	*OR*
Tenure 1	0.09	1.09	−0.32	0.73	0.40	1.50
Tenure 2	0.45 ^**^	1.57	0.60 ^**^	1.82	−0.15	0.86
Kindergarten nature	0.22	1.25	−0.02	0.99	0.23	1.26
Zone 1	0.41 ^*^	1.51	0.48 ^*^	1.62	−0.07	0.94
Zone 2	0.15	1.16	0.31	1.36	−0.16	0.86
Career adaptability	1.83 ^***^	6.21	0.58 ^*^	1.78	1.25 ^***^	3.49
Organizational identification	0.61 ^**^	1.84	0.34	1.40	0.27	1.32
Positive affect	0.16	1.17	0.34 ^**^	1.40	−0.18	0.84

*N* = 1674. ^*^p < 0.05, ^**^p < 0.01, ^***^p < 0.001. Tenure 1 (1 = ≤ 3 years) and Tenure 2 (1 = ≥ 10 years) were dummy variables transformed by tenure, in which 4 ~ 9 years as the reference base. Kindergarten nature: 0 = private kindergarten; 1 = public kindergarten. Zone 1 (1 = eastern area) and Zone 2 (1 = central area) were dummy variables transformed by geographical region, in which the western area as the reference base. Profile labels: Profile 1 = High Maintenance (high presence, high search); Profile 2 = Low Persistence (medium-high presence, low search); Profile 3 = Moderate Development (medium-high presence, medium-high search); Profile 4 = Emergent Development (medium presence, medium search). The group following “VS.” serves as the reference group in the multiple regression analysis. Odds ratios represent the change in likelihood of profile membership associated with a one-unit increase on a 5-point scale for career adaptability and organizational identification, and a 7-point scale for positive affect. These values reflect the cumulative effect across the full range of each predictor and should be interpreted in the context of the original measurement scales

All three motivational states significantly predicted profile membership, but with distinct patterns. Career adaptability was the strongest and most consistent predictor. Compared to Profile 4 (Emergent Development in Calling), teachers with higher career adaptability had 11.37 times the odds of being in Profile 1 (High Maintenance in Calling; *OR* = 11.37, *p* < 0.001), 3.26 times the odds for Profile 2 (Low Persistence in Calling Keep Unchanged; *OR* = 3.26, *p* < 0.001), and 1.83 times the odds for Profile 3 (Moderate Development in Calling; *OR* = 1.83, *p* < 0.001). Compared to Profile 3, teacher with higher career adaptability had 6.21 times the odds of being in Profile 1 (*OR* = 6.21, *p* < 0.001) and 1.78 times the odds for Profile 2 (*OR* = 1.78, *p* = 0.011). They also had 3.49 times the odds of belonging to Profile 1 than to Profile 2 (*OR* = 3.49, *p* < 0.001). The large odds ratios for career adaptability (e.g., *OR* = 11.37 for Profile 1 vs. Profile 4) reflect the cumulative effect of a one-unit increase on a 5-point scale. Given that career adaptability scores in this sample ranged from approximately 2.2 to 5.0 (*M* = 4.22, *SD* = 0.52), the observed *OR*s indicate that even modest increases in adaptability are associated with substantially higher likelihood of membership in more adaptive calling profiles.

Organizational identification and positive affect showed more selective effects, primarily distinguishing Profiles 1 and 2 from Profiles 3 and 4. Higher organizational identification increased the odds of being in Profile 1 versus Profile 4 (*OR* = 2.31, *p* < 0.001), Profile 2 versus Profile 4 (*OR* = 1.76, *p* = 0.043), and Profile 1 versus Profile 3 (*OR* = 1.84, *p* = 0.002). Higher positive affect increased the odds of being in Profile 1 (*OR* = 1.22, *p* = 0.041) and Profile 2 (*OR* = 1.45, *p* = 0.002) versus Profile 4, and Profile 2 versus Profile 3 (*OR* = 1.40, *p* = 0.002). 

## Discussion

Based on the Proactive Motivation Model [41], this study used both variable-centered and person-centered approaches to examine how career adaptability (can do), organizational identification (reason to), and positive affect (energized to) predict search for and presence of calling among kindergarten teachers. The variable-centered analysis showed that each motivational state uniquely predicts the two calling dimensions, highlighting their distinct roles. The person-centered analysis identified distinct calling profiles and revealed that the three motivational states significantly differentiate profile membership. Integrating both approaches, our findings provide a more nuanced understanding of the motivational foundations of calling development. Implications for theory and practice are discussed below.

### Theoretical implications

#### Distinct profiles combining the search for and presence of calling

Our findings identify four latent calling profiles. Profiles 1, 3, and 4 differ primarily in the degree (i.e., levels) of both calling presence and search, constituting a quantitative continuum from moderate to high engagement. Interpreting level-differentiated profiles as substantively distinct categories is not merely a statistical byproduct but is theoretically and practically meaningful. Supported by our person-centered analysis of predictors, the motivational mechanisms of these three profiles are different. For instance, of the three proactive motivational states, only stronger “can do” motivation increases the likelihood of Profile 3 (vs. Profile 4); only combined gains in “can do” and “reason to” raise the likelihood of Profile 1 (vs. Profile 3); and all three motivations increase the likelihood of Profile 1 (vs. Profile 4). These profiles help to identify developmental turning points and distinct motivational patterns, enabling targeted, step-by-step career interventions.

In contrast, Profile 2 (“Low Persistence in Calling Keep Unchanged”) shows a qualitative (shape) difference, reflecting a structural misalignment characterized by a strong current experience without future-oriented pursuit. This profile reveals a key theoretical insight: while variable-centered analysis showed a moderate positive link between calling search and presence, matching the pattern seen in most teachers (over 90%; Profiles 1, 3, and 4), only person-centered analysis uncovered a subgroup for whom this link does not hold. The coexistence of these patterns resolves prior inconsistencies about the search–presence relationship (e.g. [9, 14]), . Variable-centered methods capture average trends but mask important individual differences. Thus, person-centered analysis is essential to move beyond linear assumptions and capture how calling actually varies, including cases of structural misalignment that standard approaches overlook.

The profiles support integrating deficit-correcting and life-affirming views of meaning seeking [31]. Most teachers show aligned presence and search across varying levels of intensity, reflecting a life-affirming orientation where pursuit of calling continues or grows with experienced strength. In contrast, Profile 2 reflects a deficit-correcting pattern: after achieving meaning, motivation to explore declines. Its recurrence across studies [32, 57] suggests it is a meaningful, albeit less common, developmental path, challenging linear models of calling. Research shows this profile is linked to lower work meaning and innovation compared to high presence–high search types [32, 57], highlighting the need to understand factors driving disengagement within person-centered frameworks.

These profiles align with and extend prior research. The four-profile structure resembles Li et al.,’s [32] findings in kindergarten teachers. This replication supports the robustness of these patterns in teaching. Compared to Zhou’s [57] study of technology professionals, key differences emerge. While both find high presence–high search profiles, teachers lack the high search–low presence profile seen in tech workers. The absence of a “high search-low presence” profile among kindergarten teachers, in contrast to technology professionals [57], warrants further consideration. Several explanations may account for this occupational difference. First, teaching is inherently relational and purpose-infused; even novice teachers may experience meaningful moments with children that foster an incipient sense of calling, making pure exploration without perceived presence less likely. Second, teacher socialization processes emphasize vocational commitment and service, potentially discouraging extended periods of searching without concurrent experience of meaning. Third, the structure of teachers’ work, which involves daily immersion in caregiving and instruction, may provide continuous opportunities for meaning-making that preempt purely exploratory phases. In contrast, technology roles may offer greater distance between daily tasks and broader purpose, allowing for more prolonged searching before calling crystallizes. Future research should systematically examine how occupational characteristics—such as task meaningfulness, socialization intensity, and opportunities for purpose expression—shape the configurations of calling search and presence across professions.

#### Variable-centered perspective: unique and differential effects of proactive motivation

This study found that, consistent with the Proactive Motivation Model, career adaptability (“can do”), organizational identification (“reason to”), and positive affect (“energized to”) positively predicted teachers’ the presence of calling, with each explaining unique variance. For calling search, career adaptability and organizational identification were positive predictors, whereas positive affect was not. Career adaptability, rooted in career construction theory, is a key resource for building self-meaning and achieving a dynamic person-environment fit [44], both of which are central to calling [15]. It also functions as a meta-competency that greatly assist the person in pursuing and perceiving a calling [22]. In contrast, organizational identification fosters value alignment and embeddedness, enhancing current perception of meaning but potentially reducing motivation to explore potential alternatives and deep search. Moreover, calling search is inherently effortful and uncertain [11], sometimes triggered by negative events [40]. Positive affect reflects present satisfaction, which may encourage contentment while inhibiting exploratory behavior in the calling search stage. The lack of a unique link between positive affect and calling search challenges a direct application of the broaden-and-build theory to effortful exploration, indicating that calling search is grounded less in transient positive emotionality and more in enduring cognitive–evaluative foundations, such as perceived competence and value congruence.

Our findings reveal a critical asymmetry in the motivational underpinnings of calling: “can do” motivation (career adaptability) is the most robust, exerting equal effects on calling search and presence; “reason to” (organizational identification) and “energized to” (positive affect) motivations are more specific. These findings offer a theoretical refinement to the Proactive Motivation Model when applied to identity-based, existential goals like calling. While PMM posits that “can do,” “reason to,” and “energized to” motivations jointly enable proactive goal pursuit [41], our results suggest that these states are not functionally equivalent across all goal types. For calling—a goal centered on self-expression and meaning rather than instrumental performance—“can do” motivation (career adaptability) appears to serve as the foundational engine, providing the agentic capacity for both pursuing and experiencing purpose. “Reason to” (organizational identification) and “energized to” (positive affect) motivations, while valuable, operate more as amplifiers that enhance calling primarily when sufficient “can do” resources are already present. This suggests a hierarchical structure within the PMM framework for certain classes of goals: “can do” motivation may be a necessary condition, while “reason to” and “energized to” serve as sufficient but not necessary facilitators. Future research should test whether this asymmetry generalizes to other identity-relevant proactive goals, such as pursuing authentic leadership or crafting a meaningful career narrative.

#### Person-centered perspective: motivational predictors of calling profile membership

The person-centered analysis deepens this understanding by showing how these asymmetric motivational states jointly related to distinct calling configurations. Career adaptability demonstrated a strong and consistent positive effect on membership across all profiles. Higher career adaptability increases the likelihood of being in a higher-level profile: teachers with greater adaptability are more likely to be in Profile 3 than Profile 4, Profile 2 than Profile 3, and Profile 1 than Profile 2. This reinforces its role as a foundational “can do” motivational resource, further indicating its function as a meta-competency enabling transitions to the most adaptive calling types.

Organizational identification and positive affect demonstrated selective yet significant synergistic roles with career adaptability in predicting profile membership. First, both motivational states followed a shared pattern: when coupled with higher career adaptability, each significantly increased the odds of membership in high-presence profiles (Profiles 1 and 2) relative to the least developed baseline profile (Profile 4). Second, their divergent contributions emerged in fine-grained contrasts: organizational identification, synergizing with career adaptability, specifically elevated the likelihood of Profile 1 over Profile 3; by contrast, positive affect, synergizing with career adaptability, uniquely favored Profile 2 over Profile 3. These results confirm their positive roles in calling presence and search, but also show they are context-dependent—effective only for certain transitions and contingent on current calling status. Prior research links Profile 1 to higher job satisfaction, work meaning, and innovation; Profiles 2–4 show progressively lower levels of these outcomes [32, 57]. Our findings imply that teachers striving toward a more optimal calling profile may derive greater motivational benefit from strengthening organizational identification than from cultivating positive affect.

A critical boundary condition for this synergy is noteworthy: neither organizational identification nor positive affect adds motivational value beyond career adaptability in differentiating Profile 3 from Profile 4, or, more importantly, Profile 1 from Profile 2. This shows that “reason to” and “energized to” states do not operate independently; they may depend on a foundational level of career adaptability. Between Profile 4 and Profile 3, the overall motivational resource pool remains constrained. At this stage, boosting “reason to” or “energized to” motivation, without enough strengthen on the core “can do” capacity, fails to trigger meaningful change in calling configuration. The shift from Profile 2 to Profile 1 reflects a move from passive acceptance to active, effortful calling maintenance, enabled by high-agency self-regulation, the core of career adaptability. Neither moderate organizational identification nor positive affect in Profile 2 can substitute for the adaptive competencies required to initiate, sustain, and reflect on exploratory career behaviors. In short, organizational identification and positive affect are not primary drivers, but conditional amplifiers of career adaptability, the primary agentic resource underlying vocational agency. This proposition requires empirical validation.

Collectively, this study extends the Proactive Motivation Model by shifting focus from motivation for explicit active behaviors and performance to the motivational underpinnings of “being and becoming”. Moreover, our findings refine the PMM by demonstrating asymmetry among its three motivational states in the domain of calling. While the PMM posits their importance for proactive goals, our results suggest that “can do” motivation (career adaptability) serves as a primary and generalizable engine for calling development. “Reason to” (e.g., organizational identification) and “energized to” (e.g., positive affect) motivations, while valuable, operate more as secondary and context-dependent facilitators. Furthermore, our study advances the conceptual and practical understanding of calling by distinguishing between experiencing and pursuing a calling. These contributions directly address recent calls for research on calling search [11], challenge a uniform application of motivational theory, and support a more nuanced, pathway-sensitive understanding of calling development.

### Practical implications

Our findings provide practical implications for kindergarten teachers’ professional development by identifying both general intervention targets and profile-specific strategies. First, career adaptability emerged as the strongest and most consistent predictor of both calling search and presence across all profiles, highlighting the need to strengthen teachers’ adaptive capacity. Institutions should integrate adaptability training into in-service programs. For example, virtual reality simulations can replicate emerging educational contexts or potential career challenges, such as organizing activities or managing parent-teacher communication in the context of AI-assisted instruction. Such experiential training not only strengthens teachers’ ability to adjust pedagogical strategies flexibly but also enhances self-regulation and professional resilience in the face of evolving educational demands.

Second, organizational identification significantly predicts both the presence and pursuit of calling, though its influence is more selective than career adaptability’s. To strengthen teachers’ emotional and psychological ties to their institutions, schools should involve them in co-creating missions, strategic decisions, and aligning personal goals with institutional vision. This fosters ownership and belonging, transforming kindergartens from transactional workplaces into purpose-driven communities.

Third, positive affect is linked primarily to calling presence, underscoring the value of emotionally supportive environments. School leaders should create structured opportunities, such as reflection sessions or peer recognition—for sharing positive classroom experiences. To ensure these emotions contribute to lasting vocational purpose, guided reflection is essential. Workshops that help teachers connect meaningful moments—like a child’s developmental breakthrough—to their core values can bridge fleeting positivity with sustained calling.

Finally, the distinct calling profiles identified in this study highlight the limits of one-size-fits-all training and call for differentiated, profile-sensitive interventions. For teachers in Profile 4 (Emergent Development) characterized by moderate levels of both search and presence, comprehensive foundational support is needed. Interventions should simultaneously strengthen career adaptability (e.g., through scenario-based training), organizational identification (e.g., through value-clarification exercises), and positive affect (e.g., through peer recognition programs) to catalyze movement toward more developed calling configurations. For teachers in Profile 3 (Moderate Development) who show medium-high presence and search, efforts should focus on consolidating gains and preventing stagnation. Enhancing career adaptability remains critical, but organizational identification becomes increasingly important. Mentoring programs that connect these teachers with experienced colleagues can deepen their sense of belonging and purpose. For teachers in Profile 2 (Low Persistence) who experience calling but have ceased exploration, re-engagement strategies are needed. Career adaptability training focused on “curiosity” and “confidence” dimensions may help reactivate exploratory motivation. Structured reflection on how calling might evolve with changing circumstances could transform passive acceptance into active maintenance. For teachers in Profile 1 (High Maintenance) who demonstrate optimal calling, interventions should focus on sustainability and preventing burnout. Leadership opportunities, such as mentoring others or participating in curriculum innovation, can channel their vocational energy into generative contributions that reinforce rather than deplete their calling.

### Limitations and future research

Several limitations should be acknowledged, each suggesting directions for future research. First, our cross-sectional design limits causal inference about the relationship between proactive motivation and calling. Specifically, we measured positive affect over the past three months, whereas calling is typically viewed as a relatively stable orientation. This temporal mismatch between our measure of positive affect and the conceptualization of calling as a relatively stable orientation points to important directions for methodological advancement. First, experience-sampling methods (ESM) could examine whether daily fluctuations in positive affect predict same-day variations in calling salience, and whether accumulated positive affective experiences over weeks predict gradual shifts in calling perception. Second, longitudinal cross-lagged panel designs with multiple waves (e.g., 3–4 time points over 1–2 years) could test whether sustained positive affect precedes calling development, whether calling enhances positive affect, or whether the relationship is reciprocal. Third, measurement burst designs—combining daily diaries within annual assessments—could disentangle state-like fluctuations from trait-like changes in both positive affect and calling. Such designs would clarify whether positive affect functions as an antecedent, concomitant, or consequence of calling development, addressing a critical gap in understanding the temporal dynamics of vocational purpose.

Second, this study demonstrated each motivational state’s distinct predictive utility and did not test their potential dynamic interplay (e.g., “reason to” and “energized to” motivation may foster “can do” motivation; [41]). Investigating such relationships represents a more integrative application of the PMM and a promising direction for future research using moderated or mediated designs. Future studies could also extend our work by examining alternative valid measures of these states (e.g., role-breadth self-efficacy, autonomous motivation, work passion) and the indirect effects of distal antecedents (e.g., leadership, organizational climate) specified by the PMM. Additionally, future research should operationalize motivational constructs into observable behaviors to bridge theory and practice. Ahmadi et al., [1] empirically show that teachers’ motivational behaviors, particularly those satisfying students’ autonomy, competence, and relatedness needs, effectively foster intrinsic learning motivation and improve learning outcomes. Building on Ahmadi et al., [1], we propose an evidence-based taxonomy of motivational behaviors that cultivate teachers’ “can do”, “reason to”, and “energized to” states. Such a behaviorally grounded taxonomy would provide a science-informed blueprint for targeted professional development, such as coaching programs for kindergarten leaders or mentor training, to strengthen the proximal motivational conditions underlying teachers’ sense of calling.

Third, this study examined search for and presence of calling but not living a calling. Prior research shows a strong correlation between perceiving and living a calling among Chinese kindergarten teachers (*r* = 0.80; [35]), justifying the exclusion of the living dimension to avoid multicollinearity. Future studies should develop integrated models assessing all three calling components. These models could clarify whether strong calling presence translates into effective enactment and whether enactment challenges reignite calling search. Furthermore, search for and presence of calling are multidimensional, reflecting both self-oriented (e.g., purpose, meaning) and other-oriented (e.g., duty, social contribution) aspects [9, 12], which link differently to well-being [39]. Future research should employ multidimensional measures to replicate this study’s findings. Such multi-faceted calling measures also allow us to examine whether different combinations of these calling dimensions emerge across distinct populations and investigate the antecedents and consequences of specific calling dimensions separately and their combinations.

Finally, focusing exclusively on Chinese kindergarten teachers limits generalizability. Future research should test whether similar calling profiles and motivational patterns appear across diverse cultural, educational, and occupational contexts to strengthen cross-contextual validity.

## Conclusion

This study integrates variable-centered and person-centered approaches to examine how proactive motivational states relate to kindergarten teachers’ calling. The variable-centered results show that career adaptability, organizational identification, and positive affect, as three distinct forms of proactive motivation, exert unique and differential effects on presence of and search for calling. The person-centered analysis identifies four calling profiles based on varying levels of the search and presence dimensions of calling, revealing that three motivational states function differently across calling profiles. Career adaptability consistently distinguishes all profiles, serving as a core resource, while organizational identification and positive affect serve more selective roles. These findings support the proactive motivation model and capture the complexity of motivational influences on vocational experiences.

## Data Availability

The data presented in this study are available upon request from the corresponding or the first author.
